# Identifying and quantifying membrane interactions of the protein human *cis*‐prenyltransferase

**DOI:** 10.1002/pro.70167

**Published:** 2025-05-24

**Authors:** Duncan M. Boren, Shiri Kredi, Ekaterina Positselskaya, Moshe Giladi, Yoni Haitin, Josh V. Vermaas

**Affiliations:** ^1^ MSU‐DOE Plant Research Laboratory and Department of Biochemistry and Molecular Biology Michigan State University East Lansing Michigan USA; ^2^ Department of Physiology and Pharmacology, Faculty of Medical and Health Sciences Tel Aviv University Tel Aviv Israel; ^3^ Tel Aviv Sourasky Medical Center Tel Aviv Israel; ^4^ Sagol School of Neuroscience Tel Aviv University Tel Aviv Israel

**Keywords:** free energy perturbation, FRET, molecular dynamics, prenyl synthesis, protein‐membrane interaction

## Abstract

Prenyl chains come in multiple sizes, fulfilling unique and essential functions across all domains of life. Prenyl chains are synthesized by prenyltransferase proteins. Despite their structural similarity, prenyltransferases exhibit substantial functional diversity to create lipophilic products of varying lengths. Human *cis*‐prenyltransferase (h‐*cis*PT) is a tetrameric enzyme responsible for the synthesis of long prenyl chains, consisting of 20‐prenyl‐unit products that are essential to specific posttranslational modifications such as N‐glycosylation upon downstream processing. These long products are hypothesized to transfer from h‐*cis*PT to the ER membrane, but the mechanism of this transfer is not known. We use molecular dynamics simulations to identify a consistent membrane binding pose for h‐*cis*PT. By quantifying protein‐membrane contacts, we identify the aromatic amino acid residues in the conserved catalytic domain as critical to membrane binding. Determining relative protein‐membrane binding free energies through free energy perturbation highlights the importance of these residues for membrane association, as mutations lower membrane affinity by as much as 27 kcal/mol. These results are validated using FRET to demonstrate decreased catalytic activity and membrane binding in response to mutation. Together, our results suggest a possible mechanism for prenyl substrate transfer, where key aromatic residues facilitate h‐*cis*PT binding to the ER membrane in an orientation that holds the substrate‐containing active site near the membrane surface. Molecular dynamics simulations of the mutant exhibiting lower FRET show greater orientational variability relative to wild type. This evidence for a specific orientation of h‐*cis*PT provides a structural basis for isoprenoid association to the membrane during synthesis and prior to its release.

## 1 INTRODUCTION

Isoprene is a key building block for biological polymers across all domains of life (Holstein and Hohl 2004; Matsumi et al. 2011). Isoprene is a gas with signaling roles in plants (Kulke et al. 2023a; Sharkey et al. 2007; Weraduwage et al. 2024), but isoprene polymers have many bioactive forms with diverse functions across biology. Prenyl polymers are used to prenylate specific proteins on biological membranes (Wang and Casey 2016), and are precursors to sterols and steroids (Du et al. 2022; Nes 2011), are integrated into other membrane‐associated co‐factors such as quinones (Kawamukai 2018; Nowicka and Kruk 2010), and facilitate protein glycosylation (Grabińska et al. 2016). Polyprenes come in multiple sizes, synthesized by the appropriate prenyltransferase enzyme (Chen et al. 2020; Yamashita and Takahashi 2020). Rubber is a very long natural polyprene with wide industrial application, while other isoprene polymers may only be 10–15 carbons (Chen et al. 2020).

Polyprenes generated by prenyltransferases can be either *cis*‐ or *trans*‐ with respect to a 1,4‐alkene bond (Christianson 2017). Despite the disparate size of the end products, the *cis*‐prenyltransferase enzymes found across biology share structural features (Edani et al. 2020). Prokaryotic *cis*‐prenyltransferases (*cis*PTs) are well characterized in structure and function (Kharel et al. 2006; Noike et al. 2008), and tend to generate shorter isoprenoid products that fit into the hydrophobic pocket near the enzyme active site, creating a clear mechanism to regulate product size. By contrast, eukaryotic *cis*PTs generate much longer polyprenes that cannot fit into the active site and hydrophobic pocket within the protein (Kharel et al. 2006).

**FIGURE 1:**
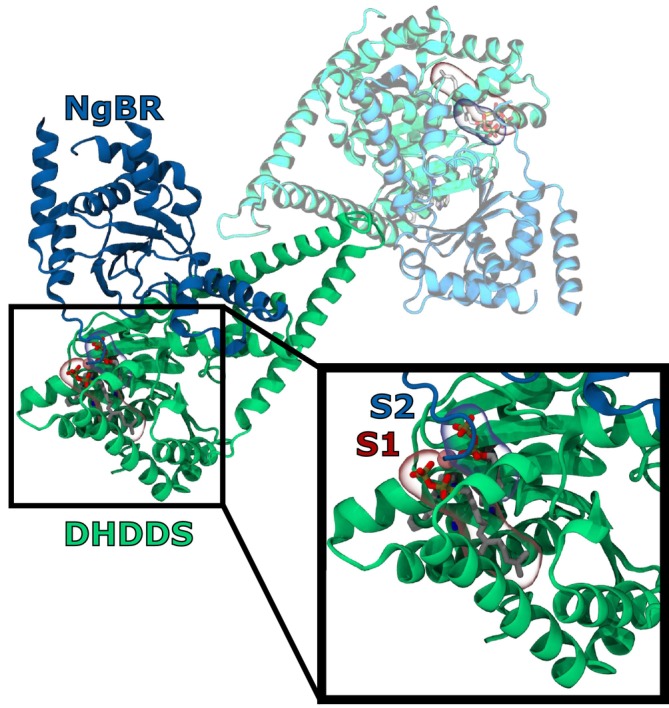
h‐*cis*PT is thought to assemble into a tetrameric complex of two heterodimers (PDB: 7PB1), combining DHDDS together with NgBR. As each heterodimer within the tetramer is identical, we are focusing our simulation efforts on a single heterodimer, represented here in darker shades of blue and green. Recent structures have also had prenyl substrate present, in this case GGS (Giladi et al. 2022), represented here with spheres for the resolved heavy atoms (gray for carbon, red for oxygen, yellow for sulfur, and brown for phosphorus).

The heterotetrameric human *cis*‐prenyltransferase (h‐*cis*PT), which synthesizes the precursor for dolichol (Edani et al. 2020), is composed of two identical heterodimers (Bar‐El et al. 2020) (Figure 1). One subunit, dehydrodolichyl diphosphate synthase (DHDDS), contains the catalytic site for prenyl chain extension (Harrison et al. 2011). The catalytic site contains an active site 1 (S1), which contains the growing prenyl chain, while active site 2 (S2) binds isopentenyl diphosphate (IPP), a five‐carbon building block which is iteratively added to the prenyl chain. Past research shows that h‐cisPT membrane association is strongly linked to prenyl substrate extension, and DHDDS may be able to independently associate with the membrane (Edani et al. 2020; Giladi et al. 2022). The other subunit, Nogo‐B receptor (NgBR), lacks catalytic residues and is thought to contribute to the active site architecture (Giladi et al. 2022; Harrison et al. 2011) or potentially other interaction partners (Park et al. 2016). The N‐terminus of NgBR contains a transmembrane helix responsible for attaching the protein to the ER membrane and a long, flexible region linking the protein complex and the transmembrane domain (Harrison et al. 2011).

Long prenyl chains are released from h‐*cis*PT when they reach a length of 17–20 isoprene units, or around 100 carbons (Bar‐El et al. 2020). Such a long prenyl chain would be highly hydrophobic and very unlikely to dissociate from h‐*cis*PT in solution. This implies that h‐*cis*PT must associate with the membrane for product release and may be frequently associated with the membrane (Giladi et al. 2022). Through molecular simulation and molecular biology techniques, we determine the binding pose for the soluble domains of h‐*cis*PT to ER membranes during synthesis and investigate potential downstream impacts on product release.

## 2 METHODS

The methods used here are a combination of simulation and complementary molecular biology experiments to test predictions made by simulation and validate conclusions. To facilitate following the flow, the methods are interwoven by subsection.

### 2.1 Equilibrium system assembly

To develop a simulation model for the membrane‐bound state of h‐*cis*PT, we begin with a previously determined X‐ray crystallography structure (PDBID: 6Z1N) (Bar‐El et al. 2020). We make several alterations from this starting point. The crystal structure omits the first 75 residues of the protein, which constitute a transmembrane domain (Bar‐El et al. 2020) and flexible linker region. The transmembrane domain is located at the N‐terminus of the NgBR domain, and a flexible helix makes up the remaining residues between the transmembrane domain and the residues present. Due to the length of this linker region, the transmembrane segment is not a rigid restraint on the binding orientation for the catalytic DHDDS. Further, truncation of the NgBR N‐terminus has not been shown to affect enzyme function in previous in vitro experiments (Bar‐El et al. 2020; Edani et al. 2020; Giladi et al. 2022). The crystal structure is also missing a loop, stretching from residue 167 to 175. Using the amino acid sequence and RosettaFold (Baek et al. 2021), we have modeled the loop in our simulated structures. We elect to use the heterodimer structure, rather than a heterotetramer, as the tetrameric structure of h‐*cis*PT is still somewhat ambiguous. Based on our proposed heterotetramer structure (Figure 1) based on the published 6Z1N structure, it is not feasible for both DHDDS subunits to simultaneously associate with a planar membrane. While h‐cisPT has several possible glycosylation sites as determined by glycosylation prediction tools (Gupta and Brunak 2002; Steentoft et al. 2013), most are uncertain ‐ particularly those in the catalytic DHDDS domain, which is most closely associated with membrane binding‐related substrate release. It is important to note that h‐*cis*PT is functional even when expressed in heterologous systems where glycosylation patterns would be different (Edani et al. 2020; Grabińska et al. 2017; Shridas et al. 2003), and so glycosylations are likely not essential to function.

We use the symmetric ER model composition determined by Pogozheva and co‐workers as the starting point to model the human ER (Pogozheva et al. 2022), assembling the system in CHARMM‐GUI (Jo et al. 2008; Jo et al. 2009; Pogozheva et al. 2022). This membrane contained a combination of phospholipids, sulfolipids, and cholesterol, of varying tail lengths, with the final composition and lipid charge quantified in Table 1. Each membrane leaflet contained 190 lipids.

**TABLE 1:** Membrane composition by lipid type, based on the membrane composition for an ER membrane (Pogozheva et al. 2022).

Lipid head	Lipid tail	Count	Charge
PC	16:0/18:0	16	0
PC	16:0/18:2	18	0
PC	18:0/20:4	27	0
PE	16:0/18:1	5	0
PE	16:0/18:0	8	0
PE	18:0/20:4	7	0
PI	18:0/20:4	3	−1
PI	18:0/18:2	3	−1
PS	18:1/18:2	3	−1
SM	18:1/16:0	4	0
PA	16:0/18:1	1	−1
CHOL		5	0
Total		100	

**FIGURE 2:**
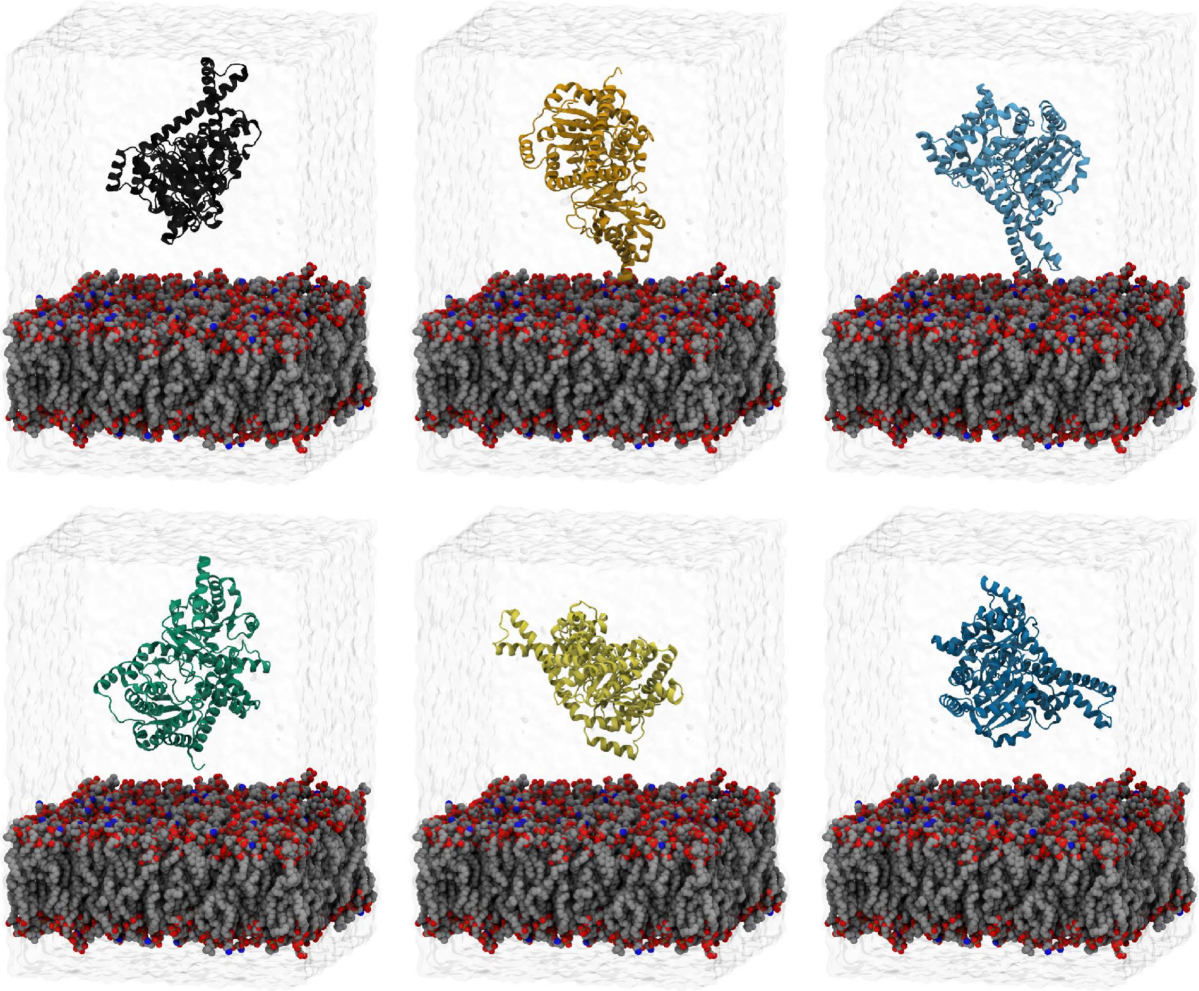
Molecular dynamics simulation setups to test membrane association (side view). The h‐*cis*PT heterodimer (cartoon) is placed above a bilayer (van der Waals) with a similar composition to the plasma membrane and solvated in water (clear surface). Since we want the bound pose to be as unbiased as possible, the proteins in each structure have a different face toward the membrane surface initially.

From this common membrane, six unique simulation systems were constructed by placing a dimer with a DHDDS and a NgBR subunit above the membrane. We simulate the dimer rather than the tetramer here (Figure 1), as pilot simulations indicated that rotational diffusion for the tetramer was very slow relative to achievable simulation timescales. The protein structure was placed 30 Å above the membrane surface and rotated in increments of 90 degrees such that each of the six simulation systems (replicas) had a different face of the protein oriented towards the membrane (Figure 2). This protocol has been used previously in the literature to arrive at consistent binding orientations for peripheral membrane proteins (Arcario et al. 2011; Kulke et al. 2023b). A 30 Å distance was selected so that the protein would have ample room to rotate before contacting the membrane. After rotation, the system was solvated and ionized using the VMD (Humphrey et al. 1996) SOLVATE and AUTOIONIZE tools, called via custom‐written python scripts. Following solvation, the system had approximately 200,000 atoms. The final simulation box has dimensions of 10 nm by 10 nm by 15 nm. This size was chosen to permit realistic protein dynamics without periodic boundary effects while optimizing simulation speed.

### 2.2 Molecular dynamics simulations

The systems described above were briefly minimized and equilibrated using NAMD 2.14 to eliminate bad contacts. Simulations were performed in NAMD 3.0a9 (Chen et al. 2020; Phillips et al. 2020) using explicit solvent and CHARMM36 carbohydrate, lipid, and protein force fields (Guvench et al. 2011; Huang et al. 2017; Klauda et al. 2010; Phillips et al. 2020). In keeping with CHARMM standard, a 12 Å cutoff was used, together with the TIP3 water model (Jorgensen et al. 1983). Long range electrostatics was handled using the particle mesh Ewald method with 1.2 Å grid spacing (Darden et al. 1993; Essmann et al. 1995). The system was simulated in an NPT ensemble using a Langevin thermostat and barostat (Feller et al. 1995). The barostat decoupled the membrane normal axis from the membrane plane, creating semi‐isotropic pressure coupling. To enable 2 fs timesteps, we used the SETTLE algorithm to fix hydrogen bond lengths (Miyamoto and Kollman 1992). Unless otherwise stated, systems were simulated to a length of 1 μs.

### 2.3 Substrate extension simulations

The prenyl product present in the crystal structure (Giladi et al. 2022) is substantially shorter than the mature natural product, featuring only 20 carbons rather than 100 (Bar‐El et al. 2020; Giladi et al. 2022). To facilitate investigations into the substrate egress pathway, we extended the prenyl ligand to a mature length by carrying out sequential alchemical additions of prenyl units to the end of the chain, starting from the most promising bound state determined from equilibrium simulation (replica 3).

The initial 20‐carbon prenyl substrate (phosphonooxy‐[(10E)‐3,7,11,15‐tetramethylhexadeca‐2,6,10,14‐tetraenyl]sulfanyl‐phosphinic acid, GGS for short), was fit into the active site by alignment to the original crystal structure, after brief simulation using NAMD 2.14 to eliminate bad contacts and evaluate the parameter performance. GGS parameters were developed using CGenFF (Vanommeslaeghe et al. 2010; Yu et al. 2012). To extend the prenyl chain, an additional prenyl unit was added to the end of the product using psfgen, and an alchemical simulation was performed over 50 ns to gradually add the additional 5 carbons to the end of the tail into the S1 site while allowing the growing chain to be flexible within the S1 pocket. Extensions were run repeatedly until the ligand was 20 prenyl units (100 carbons) in length, corresponding to the length of a mature substrate in‐vivo. An additional five simulations were run using the fully extended product as a starting point, each for 1 μs using the same protocol as above.

To monitor what conformations are plausible for a membrane‐embedded prenyl chain with intermediate length that still attached to h‐*cis*PT, independent simulations were carried out where the extension was incomplete (9 prenyl units). After extension, an intermediate length substrate was found to occupy the interface between aqueous solution and the membrane. From this starting position, five additional replicas were simulated for an additional 1 μs to sample protein‐membrane orientations on the membrane for intermediate product lengths. To determine the capacity of shorter chains to form sustained interactions with the ER membrane, simulations were carried out with a 15‐unit prenyl chain, which is long enough to contact the membrane surface, but shorter than the mature products produced by h‐*cis*PT. Simulations started from a position where the prenyl chain was in contact with the membrane. From this position, five simulations were carried out for a total of 250 ns.

### 2.4 Equilibrium mutant simulations

As will be shown in results, two specific residues, W238 and F241, were predicted to be important for membrane association for DHDDS to the membrane. To determine the role played by these residues in protein‐membrane interactions, the W238AF241A double mutant was developed in psfgen. From the same starting pose as the ligand‐present simulations (the end of replica 3 from the initial apo simulations), 5 simulation replicates were run to 1 μs to assess the impact on binding and dynamics for h‐*cis*PT.

### 2.5 Free energy perturbation simulations

Multiple residues were found to bind to the membrane. To quantify the significance for individual residues in protein‐membrane interactions, we used free energy perturbation (FEP) simulations to determine the relative affinity of multiple mutants to the membrane when compared to the wild type. Four aromatic residues in the binding region stood out for mutation to alanine or aspartate—W12, F15, W238, and F241. Alanine mutants were developed specifically to test the importance of individual amino acids in the binding, while aspartate mutants probed the impact of charge. Several double mutants were also tested to investigate if combining mutants would result in a greater than additive increase in binding free energy. The full set of mutants tested is W12A, W12D, F15A, F15D, W238A, W238D, F241A, F241D, W12AW238A, W12DW238D, F15AF241A, F15DF241D, W238AF241A, and W238DF241D. A custom python script was used to create dual topology structures within VMD.

In perturbation simulations, lambda steps between 0.0, 0.02, 0.05, 0.1, 0.2, 0.3, 0.4, 0.5, 0.6, 0.7, 0.8, 0.9, 0.95, 0.98, 1.0 were used, with 3 ns of simulation used for each lambda value, for a total of 45 ns per alchemical transition, and 90 ns per reaction. To prevent the protein from drifting off the membrane surface due to low natural affinity, harmonic constraints were applied to membrane phosphates as well as the alpha carbons of h‐*cis*PT during the FEP simulation so that the bound state is preserved, even if the interaction is unfavorable. The potential energy function of the harmonic restraint is $U\left(x\right)=k{\left(x-x_{\mathrm{ref}}\right)}^{2}$ where *k* is 1 kcal/mol Å^2^ and $\left(x-x_{\mathrm{ref}}\right)$ is the distance between the alpha carbon and its reference position. Equivalent transformations were conducted for the protein in solution to complete the thermodynamic cycle. In bound state simulations, the energy involved in maintaining restraints tended to increase while moving from wild type to the mutant state. Energy associated with protein restraints was recorded at each timestep, and can be seen in Figures S3 and S4, Supporting Information highlighting how consistent the applied restraints are across different values for λ.

**FIGURE 3:**
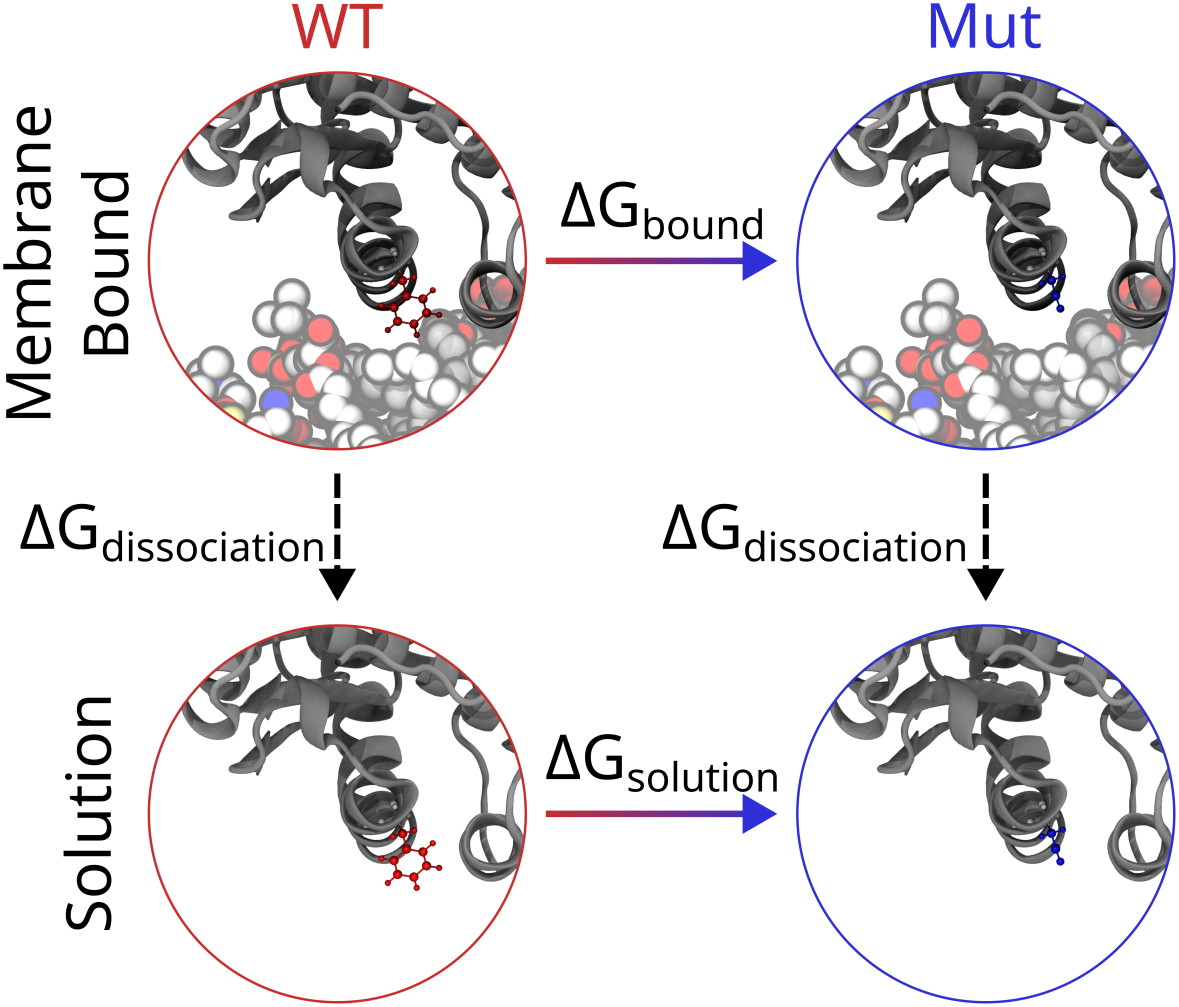
FEP thermodynamic cycle. A visual depiction of the thermodynamic cycle used to calculate the effect of an F241D mutant on membrane binding affinity, standing in for all other mutants. For each mutant, we determine the binding free energy difference for the horizontal legs connecting bound and solution states (Equation (1)). In this representation, wild‐type proteins are on the left and are circled in red, while mutants are on the right and circled in blue, so $\Delta G_{\text{bound}}$ and $\Delta G_{\text{solution}}$ follow the solid arrows as indicated from red to blue. In FEP, the dashed arrows represent different hydration free energies for the wild‐type and mutant proteins, and are not calculated, but complete the thermodynamic cycle, as the sum for the free energy changes around the cycle must be zero.

The free energy difference along each studied process was analyzed by ParseFEP (Liu et al. 2012) (Figure 3). We determine ΔG for the mutation both when the protein is bound ($\Delta G_{\text{bound}}=G_{\text{bound}}^{\text{mutant}}-G_{\text{bound}}^{\mathrm{WT}}$). The equivalent calculation for the solution system, $\Delta G_{\text{solution}}=G_{\text{solution}}^{\text{mutant}}-G_{\text{solution}}^{\mathrm{WT}}$ sets up the following calculation for ΔΔG, the relative change in binding free energy,

(1) $$\Delta \Delta G=\Delta G_{\text{bound}}-\Delta G_{\text{solution}}.$$

Negative ΔΔG values correspond to a tighter binding for the mutant protein to the membrane, while positive ΔΔG values represent a decrease in binding upon mutation.

### 2.6 Cloning

The human DHDDS (residues 1–333, UniProt Q86SQ9) and the soluble domain of NgBR (residues 73–293, UniProt Q96E22) were cloned into pET‐32b and pETM‐11, respectively. For affinity purification, the constructs include a 6×His‐tag (DHDDS) or Strep‐tag‐thioredoxin (TRX) fusion, and a TEV‐protease (tobacco etch virus) cleavage site for subsequent removal of the tags. The DHDDS W238A/D and F241A/D mutations were introduced using the NEBaseChanger tool (nebasechanger.neb.com) and verified by sequencing. Primers used for mutagenesis are listed in Table S1.

### 2.7 Protein expression and purification

The h‐*cis*PT and its mutants were expressed and purified as previously described (Bar‐El et al. 2020; Giladi et al. 2022). Briefly, *Escherichia coli* T7 xpress competent cells were co‐transformed with DHDDS and NgBR. The cultures were grown in Terrific Broth medium at 37°C until reaching OD_600nm_ = 0.6. Protein expression was induced at 16°C by adding 0.5 mM isopropyl *β*‐D‐1‐thiogalactopyranoside (IPTG), and the cells were cultured under these conditions for 16–20 h. Cells were then collected by centrifugation a (5700*g* for 15 min) at 4°C and resuspended in a lysis buffer containing 20 mM HEPES (pH 7.5), 150 mM NaCl, 1 mM tris(2‐carboxyethyl)phosphine (TCEP), and 0.02% (w/v) Triton X‐100, supplemented with DNase I (1 μg/ml) and a protease inhibitor cocktail. The cells were homogenized and lysed using a microfluidizer, and the soluble fraction was isolated by centrifugation (40,000*g* for 45 min) at 4°C. Overexpressed proteins were first purified using a HisTrap HP column (capturing DHDDS‐containing complexes), followed by eluant application onto a Strep‐Tactin column (capturing the h‐*cis*PT heterocomplexes). Tags and TRX fusions were removed using TEV protease, and the protein mixture was concentrated and applied onto a Superdex‐200 16/60 gel filtration column, pre‐equilibrated with 20 mM HEPES (pH 7.5), 150 mM NaCl, and 1 mM TCEP. The purified proteins achieved a purity of over 95%, as determined by SDS‐PAGE analysis, and were flash‐frozen in liquid nitrogen and stored at −80°C until further use.

### 2.8 Nanodisc preparation

The membrane scaffold protein 1E3D1 (MSP1E3D1) was overexpressed and purified as previously described (Hagn et al. 2018). Briefly, hexahistidinetagged MSP1E3D1 was overexpressed in E. coli and purified using a HisTrap HP column, followed by tag cleavage using TEV protease overnight at 4°C. Subsequently, the cleaved protein was subjected to size exclusion chromatography using Superdex‐75 16/60 gel filtration column, pre‐equilibrated with nanodisc buffer, consisting of 20 mM HEPES (pH 7.5) and 150 mM NaCl. For nanodisc preparation, 0.2 mM MSP1E3D1 was mixed with 10 mM azolectin (50 mM stock, pre‐dissolved in cholate buffer, consisting of 20 mM Tris–HCl pH 7.5, 150 mM NaCl and 100 mM cholate), with or without 1% (mol/mol) 1,2‐dioleoyl‐sn‐glycero‐3‐phosphoethanolamine‐N‐(5‐dimethylamino‐1‐naphthalenesulfonyl (dansyl‐PE)), in nanodisc buffer supplemented with 100 mM cholate. The assembly mixture was then incubated at room temperature for 1 h, followed by detergent removal by stepwise incubation with 0.5 g/ml Bio‐Beads (Bio‐Rad) in two batches (1 and 3 h, respectively) at room temperature. The assembly mixture was subsequently centrifuged at 21,000*g* for 5 min and loaded onto a Superdex‐200 16/60 gel filtration column, pre‐equilibrated with nanodisc buffer.

### 2.9 Fluorescent‐based enzyme activity assay

To monitor the activity of h‐*cis*PT and its mutants, the MANT‐O‐GPP fluorescent FPP analog was used as a sensor, as described previously (Giladi et al. 2022). Briefly, purified h‐*cis*PT (0.2 μM) and MANT‐O‐GPP (5 μM) were premixed in fluorescence buffer, consisting of containing 20 mM Tris–HCl (pH 7.5), 150 mM NaCl, 10 mM *β*‐mercaptoethanol, and 0.5 mM ${\text{MgCl}}_{2}$. To initiate the reaction, IPP (100 μM) was quickly added and mixed, and fluorescence time course measurements (*n* ≥ 6 replicates) were conducted immediately in a Jasco RF‐8500 spectrofluorometer microplate reader, using Ex = 352 nm and Em = 425 nm, excitation and emission wavelengths, respectively. Data were normalized and plotted using Prism GraphPad 9.0.1.

### 2.10 FRET‐based analysis of h‐ *cis*PT membrane interaction

FRET analysis was conducted using the spectral approach established by Zheng and Zagotta (Zheng and Zagotta 2004). Briefly, FRET efficiency was quantified by exciting the donor (h‐*cis*PT tryptophans) at 280 nm and measuring emission from the acceptor (danysl‐PE‐containing nanodiscs). The acceptor emission spectrum was isolated by subtracting the normalized tryptophan emission collected from a control sample containing only h‐*cis*PT. Next, the resulting isolated spectrum was normalized pairwised to the danysl‐PE spectrum generated by direct 340 nm excitation to obtain Ratio A. To correct for direct excitation of danysl‐PE by 280 nm light, Ratio $A_{0}$ was calculated from controls containing only danysl‐PE‐labeled nanodiscs, which was then subtracted from Ratio A to yield a value directly proportional to FRET efficiency (Ratio A − Ratio $A_{0}$). Notably, the wavelength independence of FRET efficiency confirmed accurate donor component subtraction and detector linearity.

### 2.11 Analysis methodology

Analysis was conducted using Python VMD scripts built for the system with the library numpy for efficient numerics and matplotlib for plotting (Harris et al. 2020; Humphrey et al. 1996; Hunter 2007). Most analyses were conducted by analyzing intermolecular contacts present in the simulation, which measure the number of times protein residues approached the membrane within a certain cutoff distance. Depending on the cutoff distance selected, contact counts can change significantly. For our analyses, we use a weighted contact definition that allows for fractional contacts at distances near the selected cutoff. The exponentially weighted native contact definition from Shienerman and Brooks has been used in prior studies to assess membrane‐protein contacts (Sheinerman and Brooks 1998; Vermaas et al. 2018; Vermaas and Tajkhorshid 2014; Vermaas and Tajkhorshid 2017). We define contact (*C*) as

(2) $$C_{i}=\sum_{j\in B}\frac{1}{1+\exp\left(5\;{Å}^{-1}\left(d_{\mathrm{ij}}-4\;Å\right)\right)},$$

where $d_{\mathrm{ij}}$ is the distance between heavy atoms *i* and *j*, and *B* is all heavy atoms in the molecular simulation system that are not atom *i*.

Depth measurements were taken by measuring the distance along the z‐axis between the geometric center of the membrane and the nearest atom in a given amino acid residue, implemented by custom VMD (Humphrey et al. 1996) scripts written in python.

Protein tilt measurements were taken by measuring the angle between residues F15, F241, and the z‐axis. We calculate the Gibbs free energy of each tilt angle as follows:

(3) $$\Delta G=-\mathrm{kT}\;\ln\frac{P_{1}}{P_{2}},$$

where *P*_1_ is the number of frames of simulation where protein assumes a given pose, and *P*_2_ is the number of frames where it does not. We report our free energies in this case in units of kT, as we have a single molecule in our simulation system, rather than a mol of molecules where multiplying by RT would be more appropriate. This definition allows us to derive the favorability of a pose from the frequency with which it occurs in simulation.

## 3 RESULTS

### 3.1 Binding face determination

**FIGURE 4:**
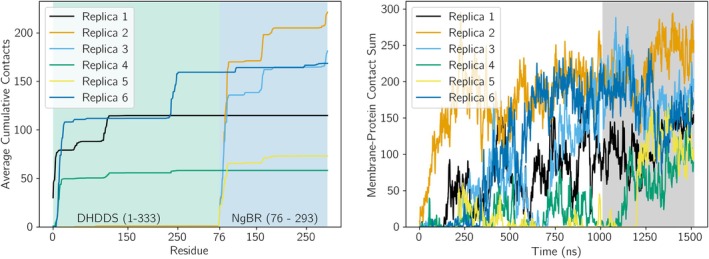
Binding pose of the h‐*cis*PT protein without a prenyl ligand. Left: Cumulative contacts between protein and membrane. The contacts between individual amino acids and the membrane are reported as a cumulative sum, to better visualize the overall effect of different protein regions on the binding interaction. The values were averaged over the last 500 ns of trajectory for each replica. A green background is used to highlight the DHDDS subunit (residues 1–333), and a blue subunit is used to highlight the NgBR subunit. Because the first 75 residues of NgBR were trimmed from our structure, NgBR contacts begin with residue 76 and proceed to residue 293. Right: Protein‐membrane contracts over time. Last 500 ns (dark gray background) were used to calculate average cumulative contacts (left).

To determine if h‐*cis*PT makes a consistent pose when binding to our ER membrane model, we analyzed six simulation replicates with different starting poses (Figure 2). We evaluate this first by quantifying the membrane‐protein contact number both over time and per residue. The h‐*cis*PT may dissociate from the membrane after initial association, as the number of protein‐membrane contacts occasionally returns (Figure 4). However, the number of protein‐membrane contacts is non‐zero over the last 500 ns of simulation time across all replicas, suggesting that this nominally soluble protein will preferentially associate with the ER membrane.

Quantifying total contacts over the whole protein is insufficient to determine a consistent binding face. Instead, we use the last 500 ns of simulation to quantify which protein residues are making specific contacts with the membrane most frequently. From 4, we see that the cumulative contacts increase rapidly in multiple regions, indicating that multiple residues make frequent contact with the membrane. Crucially, the residues making persistent contact vary across replicas (Figure 4). The most consistent interaction is the DHDDS N‐terminus, which makes substantial interactions with the membrane. In several cases, the N‐terminus of NgBR (residues 330–350 in 4) also makes strong contact. This indicates that, while h‐*cis*PT can associate with the plasma membrane prior to prenyl‐chain synthesis, multiple potential bound conformations are possible without other restraints.

**FIGURE 5:**
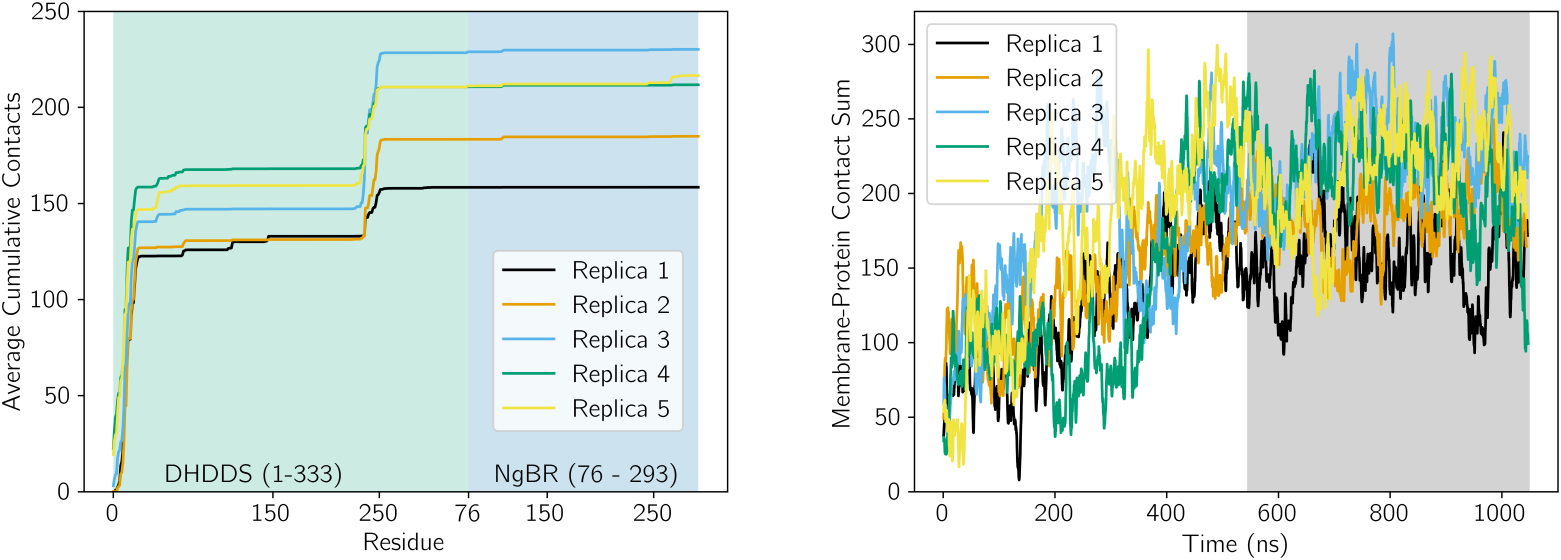
Binding pose of the h‐*cis*PT:substrate complex. Left: Cumulative contacts between protein and membrane. The contacts between individual amino acids and the membrane are reported as a cumulative sum, to better visualize the overall effect of different protein regions on the binding interaction. The values were averaged over the last 500 ns of trajectory for each replica. A green background is used to highlight the DHDDS subunit (residues 1–333), and a blue subunit is used to highlight the NgBR subunit (residues 76–293). Right: Protein‐membrane contracts over time. Last 500 ns (dark gray background) were used to calculate average cumulative contacts (left).

In addition to calculating the per‐residue average cumulative membrane contacts during simulations, we also calculated the total protein‐membrane contacts over time. Comparing the contacts‐over‐time for h‐*cis*PT before (Figure 4) and after (Figure 5) substrate insertion, we can observe a general increase in membrane contact following substrate insertion. More specifically, we observe that following substrate addition, the h‐*cis*PT protein does not dissociate from the membrane (as would be represented by contacts going to 0) in any simulations, while dissociation is observed in five out of six simulations of h‐*cis*PT prior to substrate insertion.

By evaluating the distance for the active site from the membrane, a prenyl substrate would be closest to the membrane if the final pose from replica 3 was selected for further evaluation. Other poses were judged to be implausible, as either a nascent prenyl chain would need to span a long distance in solution to reach the membrane, or the binding face would be occluded due to tetramerization. Thus, the pose from replica 3 formed the basis for further extending the prenyl product. Membrane contact analyses were repeated after the addition of an extended prenyl substrate (Figure 5).

In all five replicas from this starting point, the DHDDS N‐terminus is still responsible for most membrane contacts. However, there is also contact resulting from residues 235 through 245. The two residues with the strongest impact in this region, as measured by the number of contacts, are residues W238 and F241. These bulky aromatic residues are separated by 1 turn of an alpha helix spanning from 237 to 245. Similar bulky residues have been identified as membrane anchors in other peripheral membrane proteins (Arcario and Tajkhorshid 2014; Kulke et al. 2023b; Vermaas and Tajkhorshid 2017). The small variation in bound residues and cumulative contacts over an additional microsecond of simulation suggests that the protein‐substrate complex may occupy a stable position upon the membrane in which the N‐terminus and the 235–245 helix make two points of contact with the membrane surface during prenyl chain extension. Protein‐membrane dissociation events were not observed at any point during these simulations (Figure 5).

**FIGURE 6:**
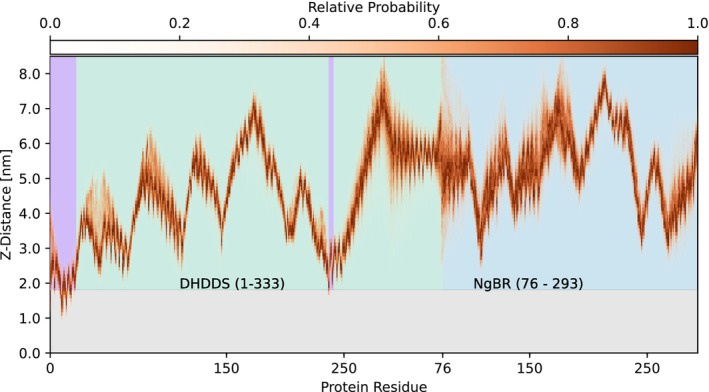
Average residue binding depth across all replicas. Residues are numbered by domain, with the first 333 residues belonging to DHDDS, followed by NgBR residues 76 through 293. Z‐distance is the distance between the center of the membrane, and the deepest non‐hydrogen atom of a selected residue. Relative probability describes the likelihood of a residue to exist at a given depth at a random time point across all simulations. Depths were collected between 500 and 1000 ns simulation time. Membrane indicated in light gray. A purple highlight has been applied to regions breaching the membrane surface.

Depth analysis (Figure 6) mirrored these results, showing membrane penetration by the first 12 residues of the protein. Beyond the N‐terminus, W238 and F241 are unique in that they may also be found beneath the membrane surface. These data indicate that the protein primarily contacts the membrane along two helices in the DHDDS domain, one stretching from L11 to K21 and the other from T236 to L245. Comparing between these two regions, the N‐terminus clearly inserts further into the membrane, with residues F15 and L11 reaching almost 1 nm beneath the membrane surface. The W238 and F241 residues, by comparison, are found to only somewhat penetrate into the hydrophobic membrane core. In this pose, the opening of the S2 active site is pointed down towards the membrane and held near the membrane surface, potentially facilitating ingress of additional IPP molecules to extend the prenyl chain.

### 3.2 Influence of binding pose

**FIGURE 7:**
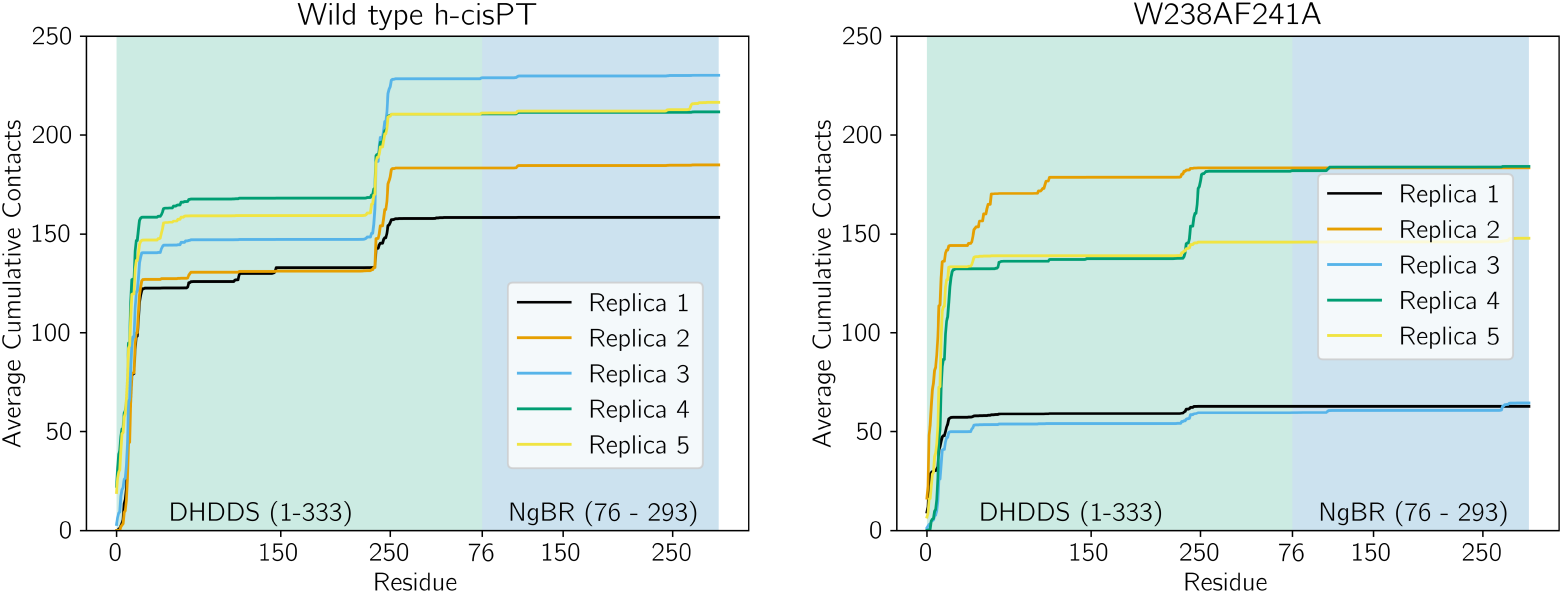
Double alanine mutant W238AF241A reduces binding. Average cumulative membrane contacts are shown between wild type (left) and W238AF241A double mutant (right) protein. The values were averaged over the last 500 ns of trajectory for each replica.

**FIGURE 8:**
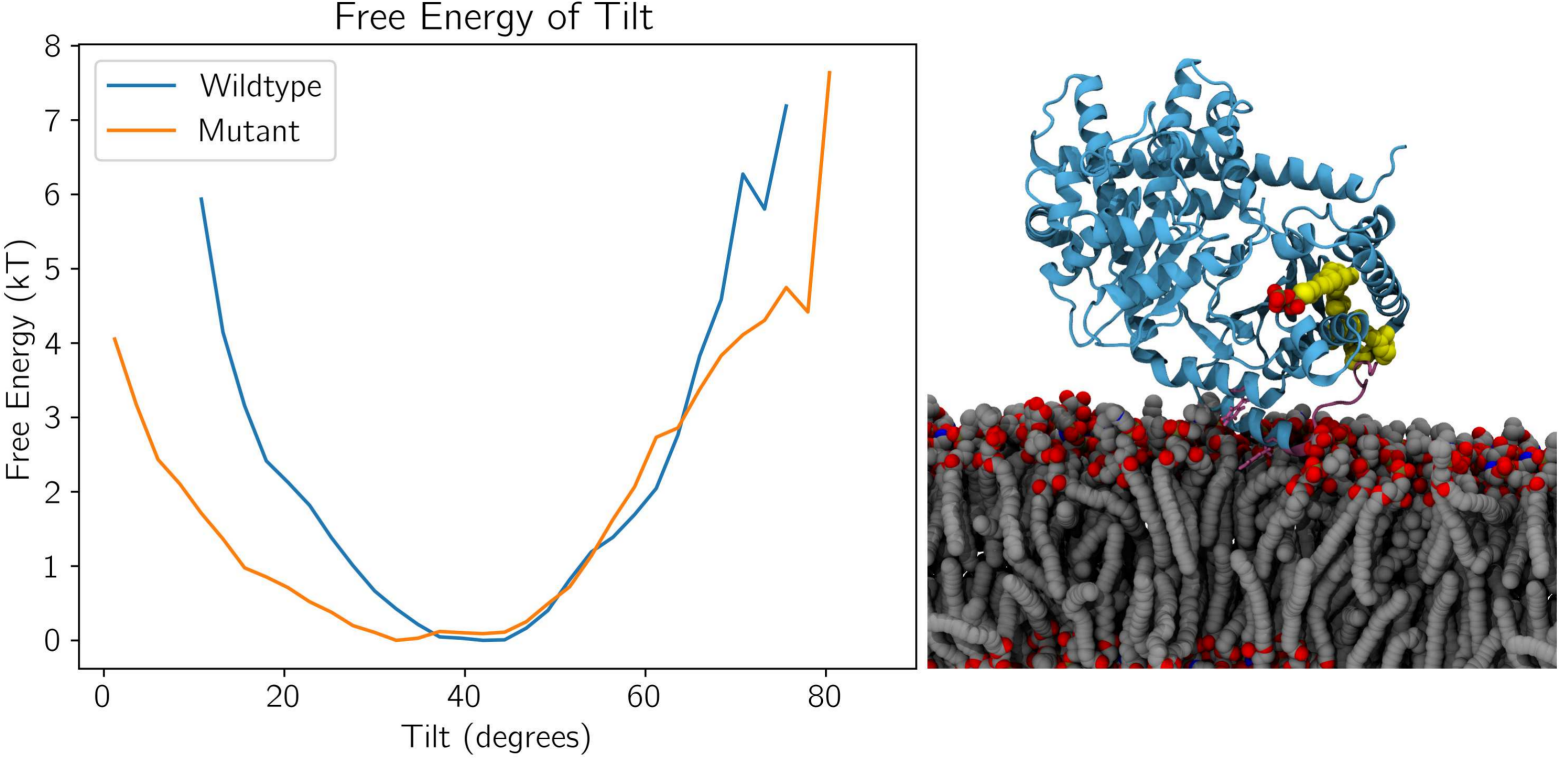
Double alanine mutant W238AF241A reduces binding pose consistency. Left: Free energies were calculated from the last half of five 1 μs simulations, based on the frequency of poses in the data. Tilt angle describes the angle formed by the vector of residues F15 and F241 intersecting with the Z‐axis. Right: The most favored tilt for wildtype h‐*cis*PT is found at 42° while the mutant is most favored at 32°. Residues F15 and F241 shown in pink sticks.

To gauge the role of the aromatic residues W238 and F241 in maintaining a stable binding pose, an alanine double mutant (W238A241A) was designed and simulated across five replicas for 1 μs simulation time. Simulations were run from the same initial state as substrate‐protein simulations. In these simulations, h‐*cis*PT does not dissociate from the membrane (Figure 5), though the residues in the neighborhood of 235–245 no longer makes consistent membrane contact. However, contact analysis of these simulations (Figure 7) shows that the reduction in membrane‐protein contacts is greater than the amount of contacts made by the 235–245 region in wild‐type proteins. This indicates that, while the h‐*cis*PT does not dissociate from the membrane, regions outside the modified helix are also making reduced contact with the membrane. Qualitative observation of simulated trajectories suggested that the mutant protein was unable to maintain a stable binding pose, rotating and swaying around the membrane‐associated n‐terminal. To quantify this change in stability of pose, we measure the distribution of the angle between the membrane normal axis and the vector connecting N‐terminus and residues 15 and 241. If the angle is 0° the vector and the membrane normal axis are co‐linear, while if the angle is 90° the vector connecting these key membrane binding residues would be orthogonal to the membrane normal, and implies that the vector is parallel to the membrane surface. Comparing the distribution of protein poses (Figure 8), the wild‐type protein spends more time occupying a narrow range of poses, while the double mutant occupies a wider array of poses across its simulations, and does not show as strong of a preference to any pose. In the wild type, the most favorable position was found at 42° tilt (Figure 8, right side), which closely resembles the binding position previously described, while the mutant's most favorable position is found at 32° tilt. This shows that the W238AF241A mutant results in a decreased stability of pose specific to the binding area of the protein, supporting the hypothesis that, although the double mutant does not result in dissociation of h‐*cis*PT from the plasma membrane, it may prevent the formation of a stable binding pose necessary for ligand insertion.

In addition to residues W238 and F241, a number of potentially significant aromatic residues on the N‐terminal domain were identified as potentially significant to protein‐membrane binding. From these residues, a series of additional single and double mutants were proposed, and wild‐type amino acids were replaced with alanine or aspartic acid. To quantify the impact of mutation, free energy perturbation calculations were used to compare the free energy associated with membrane‐binding for wild‐type and mutant proteins. All mutations tested were found to reduce protein‐membrane association relative to the wild type, with positive ΔΔG (Table 2). Among single mutants, mutations F15D and F241D were found to have the most profound negative effects (Table 2), with F15 mutations generally having the strongest effect. The greatest negative effects overall were found in double mutants F15DF241D and W238DF241D, highlighting how critical F241 is for stable membrane association.

**TABLE 2:** Free energy changes associated with protein‐membrane binding for each simulated mutation; positive free energies represent a decrease in spontaneity of protein‐membrane association.

Mutation	ΔΔG
F15A	1.37±0.07
F15D	27.7±0.5
F241A	1.73±0.07
W12A	1.86±0.13
F241D	9.18±0.22
W238A	2.74±0.11
W238D	10.0±0.4
W238DF241D	24.4±1.3
W238AF241A	3.27±0.12
W12AW238A	0.43±0.23
F15AF241A	1.85±0.07
F15DF241D	40.6±0.9

Negatively charged aspartate mutations were, in all cases, more detrimental to membrane association than alanine mutations that lack a change in charge. This is reasonable, as membrane association places residues in close proximity with negatively charged phosphate groups, which will strongly repel negatively charged residues. These results support the findings above that mutations affecting residues W238 and F241 will significantly reduce the protein's tendency to associate with the membrane. However, these are difficult calculations to do, and we anticipate that the exact values reported in Table 2 would be different had we started from a different restrained state for these restrained FEP calculations. Indeed, while the short simulations we used to determine the binding free energies are statistically accurate, with error estimates on the order of 0.1–0.3 kcal/mol, the bias introduced by selecting a specific snapshot of the trajectory to restrain coordinates means that the sampling error is likely considerably larger. Thus, rather than focus too much on the exact values, we instead think that these results are more useful as a way to gauge the relative importance of N‐terminal residues versus the W238 and F241 residues that we newly identify as having membrane contacts in this work, with F15 being the most important residue for binding, then F241, W238, and finally W12.

**FIGURE 9:**
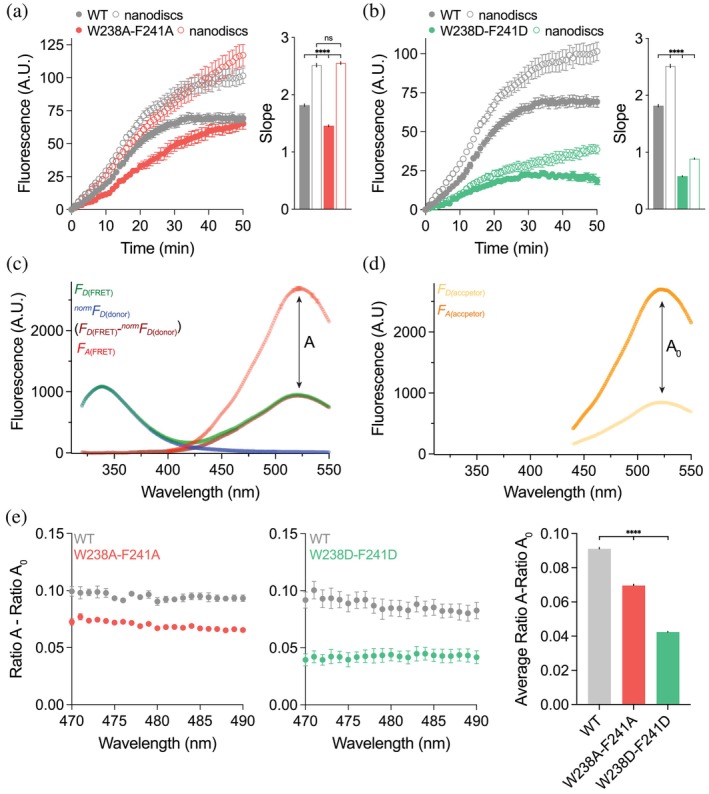
(a, b) Fluorescence‐based enzymatic activity assay, as reflected by the time‐dependent rise in MANT‐O‐GPP emission ($F_{420}$). The reaction was performed in the presence or absence of SP1D1E3 nanodiscs for WT *cis*‐PT (a, b); W238A‐F241A (a); W238D‐F241D (b). The slope, correlating with the turnover rate, was obtained from the pseudo‐linear initial reaction interval. ****p < 0.0001; *n* = 6 for each condition. A.U., arbitrary units. (c) Spectral quantification of the FRET efficiency. FRET was measured between native *cis*‐PT tryptophan residues and dansyl‐PE‐containing nanodiscs. Analysis was performed according to past work by Zheng and Zagotta (2004). Donor excited ($F_{280}$) normalized donor‐only emission spectrum (
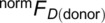
; blue line) was subtracted from FRET pair‐containing mixture spectrum ($F_{D\left(\text{FRET}\right)}$; green line) to obtain a signal composed of FRET and direct acceptor excitation ($F_{D\left(\text{FRET}\right)}{-}^{\mathrm{norm}}F_{D\left(\text{donor}\right)}$; brown line). Acceptor excited ($F_{340}$) acceptor emission spectrum ($F_{A\left(\text{FRET}\right)}$; red line) was measured from the FRET pair‐containing mixture. Ratio A represents the direct and FRET‐associated acceptor excitation of the FRET pair‐containing group. (d) Fluorescence emission spectrum of the acceptor ($F_{D\left(\text{acceptor}\right)}$ and $F_{A\left(\text{acceptor}\right)}$), following donor ($F_{280}$) and acceptor ($F_{340}$) excitation, respectively. Ratio $A_{0}$ represents the direct acceptor excitation in the acceptor‐only containing group. Subtraction of these ratios yields a pure FRET signal ($\text{Ratio}\;A-\text{Ratio}\;A_{0}$). (e) Spectral FRET (470–490 nm) between WT (gray), W238A‐F241A (pink), and W238D‐F241D (green) *cis*‐PT. ****p < 0.0001; *n* = 5–6.

To this point, the model presented is mechanistically plausible, but depends on several assumptions, such as the selected binding pose from which to extend the simulation. To validate the mechanistic hypothesis developed by simulation, double mutants of W238 and F241 were constructed and tested using fluorescent assays for their capability to extend the GGS substrate and bind to lipid bilayers. To measure chain extension, h‐*cis*PT was incubated with MANT‐O‐GPP, a fluorescent prenyl compound that increases in fluorescent intensity with chain extension and has been shown to behave analogously to natural prenyl substrates in prior research (Giladi et al. 2022; Teng et al. 2016). Fluorescence was measured in samples with and without lipid nanodiscs (Figure 9a,b). W238AF241A showed very similar fluorescence to the wild type and is not a sufficiently strong phenotype to draw firm conclusions from. By contrast, W238DF241D exhibited vastly reduced fluorescence, indicating a substantial reduction in chain extension. Further, W238DF241D mutants showed little difference in fluorescence in the presence and absence of a lipid nanodisc, suggesting that this double mutant is always in solution and cannot associate with the membrane. Förster resonance energy transfer (FRET) was used to analyze the proximity of h‐*cis*PT to a membrane. Tryptophan residues in the protein were used as FRET donors, and dansyl‐PE‐containing nanodiscs functioned as the FRET receptor. By this metric, the W238AF241A alanine mutant (Figure 9e) shows a 27% reduction in FRET signal compared to wildtype, while the W238DF241D aspartic acid mutant reduces FRET signal by 54%, indicating that aspartic acid mutants have a greater negative effect on membrane association. Thus, the experimental evidence presented in Figure 9 supports our previous FEP results, which also indicated that a W238DF241D double mutant would have a large negative impact on protein‐membrane association and that W238AF241A would have a smaller, but still negative, effect on association. Further, it provides empirical support to the claim that W238AF241A will have reduced stability in membrane association, as indicated by equilibrium mutant simulations.

## 4 DISCUSSION AND CONCLUSIONS

**FIGURE 10:**
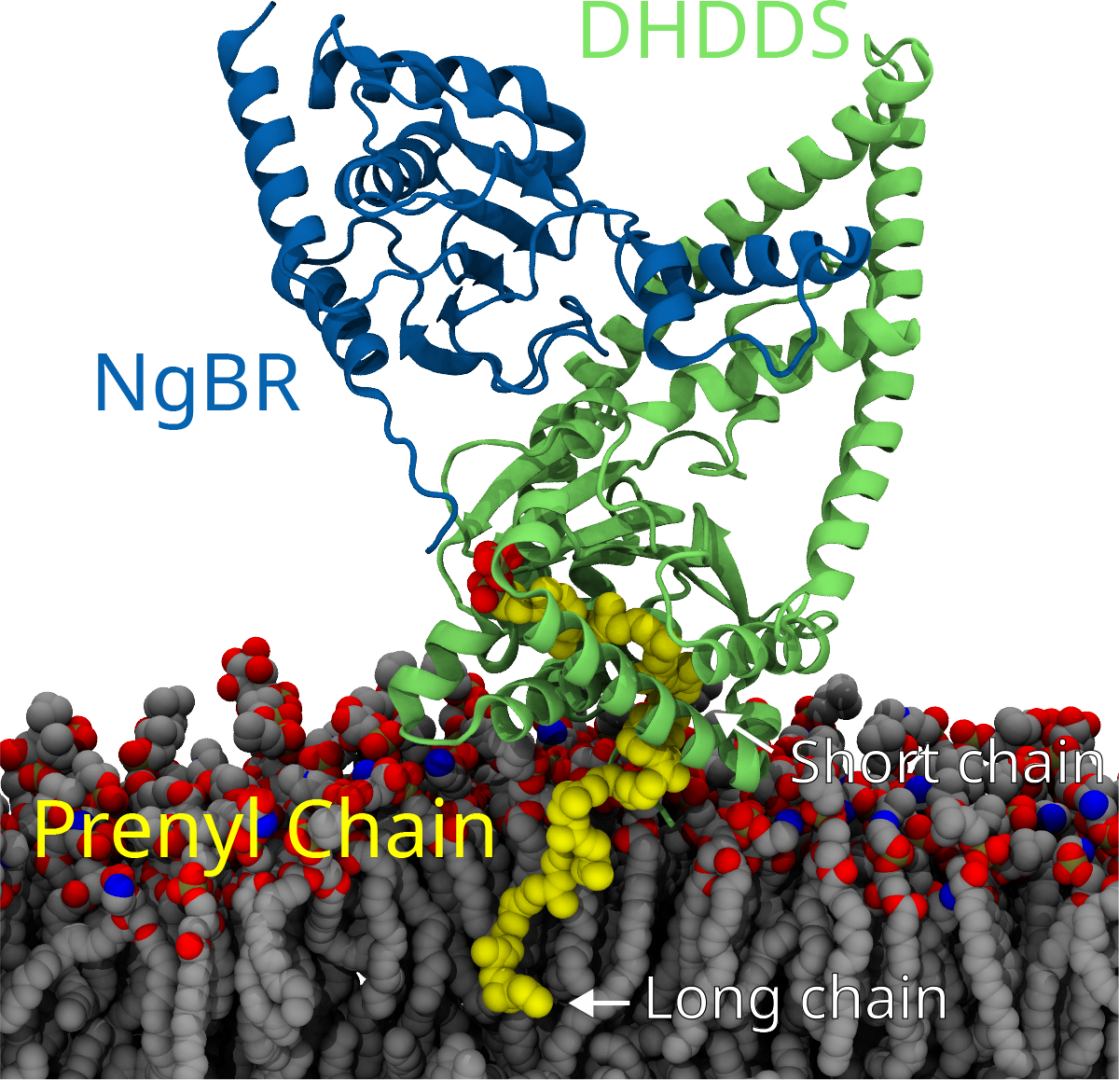
Snapshot of an extended prenyl product (dolichol) within the h‐*cis*PT. h‐*cis*PT (cartoon) synthesizes long prenyl chains, inserting a prenyl chain (yellow beads) into the ER membrane (beads colored by element, with carbons in gray, oxygens in red, phosphorus in brown, and white omitted for clarity). The h‐*cis*PT protein is heterodimeric in our simulations, with prenyl synthesis and membrane association through the DHDDS (green cartoon) subunit. NgBR (blue cartoon) is necessary for proper function in‐vivo but not catalytically active (Edani et al. 2020; Giladi et al. 2022; Park et al. 2016). *cis*PT proteins can synthesize prenyl chains at a variety of lengths, with short chains occupying the length of the active site, indicated by the white arrow, and longer chains reaching up to 20 or more prenyl units in length.

There were two primary questions to be resolved as we started this work: (1) what does the membrane association look like for h‐*cis*PT, and (2) By what mechanism does dolichol associate with and enter the ER membrane? Complementary experiments in silico and in vivo clearly answer the first question, highlighting W238 and F241 as important membrane binding residues alongside N‐terminal aromatic residues. When properly oriented, this binding face holds the active site against the membrane surface, facilitating substrate‐membrane contact, as shown in Figure 10.

While we focus on the DHDDS domain, as this is the region that clearly associates with the membrane and has such a strong phenotype if membrane interactions are disturbed, the NgBR domain also associates with the membrane. Past research has described NgBR as a multipass transmembrane domain, as well as two possible membrane‐binding helices in the NgBR N‐terminal region (Harrison et al. 2011). While the multiple putative transmembrane helices described in prior research are inconsistent with currently available structural data, there is strong evidence that NgBR has at least one transmembrane helix to anchor the protein to the ER membrane (Harrison et al. 2011). Our simulated h‐*cis*PT has a truncated NgBR N‐terminus, with the first 75 residues removed, removing any transmembrane helices, as well as the long linking helix between this transmembrane helix and the soluble domain. As a result, the lack of NgBR N‐terminus membrane association observed in our simulations is not evidence against the presence of such a TM domain, or evidence that NgBR does not substantially interact with the membrane.

This is also a place to pause and take stock of other assumptions baked into the simulation models explored here. Cleaving the N‐terminal region from the NgBR is done for computational expedience, as larger simulation volumes increase the cost of simulation. This is also why the h‐*cis*PT complex is represented as a heterodimer in our simulations, whereas the complex is believed to assemble as a dimer of heterodimers in vivo (Bar‐El et al. 2020) (Figure 1). By aligning the tetrameric crystal structure to our simulated dimer binding pose (Figure S2), we see that it is not feasible for both dimers to simultaneously associate with a planar membrane. However, the ER membrane is often highly curved, which may allow the h‐*cis*PT complex to form multiple associations (Hu et al. 2008), or even for increased spacing between lipids in the ER that might facilitate membrane binding. Alternatively, a different pairing of heterodimers from the crystallographic unit cell may represent the native protein structure. Naturally, these are all possibilities that could be explored in the future, but may depend on further advances in computing technology to lower the cost of exploring these possibilities.

Interestingly, while we do observe some association between the N‐terminus of our truncated NgBR and the membrane (Figure 4), it is from a helix stretching from residue 76 to 93 that does not map onto either of the proposed membrane binding helices. The proposed helix stretching from 117 to 135, which is included in the truncated structure, does not appear to make membrane contact. While it is perfectly feasible that the NgBR N‐terminus region could connect the protein to the ER membrane without significant orientational constraints, the reduction in membrane contact in our double mutant studies suggests that multiple membrane anchors are needed for function.

Past research has also investigated the role of mutations to the DHDDS N‐terminal. In Edani et al. (2020), h‐*cis*PT no longer increases substrate synthesis in response to PI exposure following a triple alanine mutation (W12A/F15A/I19A) affecting the DHDDS N‐terminus. This is in line with our FEP experiments, which also indicate that W12A and F15A mutations significantly reduce membrane association.

Looking at the mutation‐induced changes in binding energy in Table 2, the large decrease in binding affinity associated with aspartate mutations stands out starkly. In most cases, the alanine mutant for a given residue will have a ΔΔG 10–20 kcal/mol lower than the analogous aspartate mutant. In prior studies of moving side chain analogs through the membrane (MacCallum et al. 2008; Pogorelov et al. 2014), phenylalanine or tryptophan are favorable to insert into a membrane by about 4 kcal/mol, and alanine by about 2 kcal/mol, while aspartate is unfavorable by 10–20 kcal/mol. Thus, the energetic differences we see in Table 2 of 1–4 kcal/mol for mutations to alanine are simply a function of aromatic amino acids having more favorable membrane interactions. Likewise, mutations to aspartate are very unfavorable, as the cost of inserting a negative charge into the membrane in general is quite high, even for a transmembrane protein (Dorairaj and Allen 2007). Indeed, double mutants, though not quite additive, tend to represent more positive ΔΔG values that would indicate a weaker membrane binding interaction.

Several hints are also offered as to the mechanism by which dolichol associates with the ER membrane. We observe that the prenyl chain is capable of interfacing with the ER membrane at an immature length (Figure 10). This indicates that confining the product inside the hydrophobic pocket until release, as is the case in prokaryotes, is not a viable model for h‐*cis*PT. It has been speculated that the extension of the prenyl substrate may lead to a conformational shift in the DHDDS N‐terminus, as the growing prenyl chain pushes the N‐terminus away from the opening of the DHDDS active site (Giladi et al. 2022). There is some support for this in our simulations, as adding a prenyl substrate to the S2 active site results in the N‐terminus making stronger contact with the ER membrane across all simulations and becoming the highest‐contact region of the protein (Figure 5). Once associated with the ER membrane, the N‐terminus appears unable to induce the prenyl substrate's exit from the binding pocket. This suggests that immature prenyl products may be able to form and maintain membrane contacts, as was observed in an intermediate‐length prenyl product chain. Further, in simulations with a mature dolichol, the dolichol is inserted into both the membrane and h‐*cis*PT (Figure 10).

To summarize, our experiments identify a preferred binding pose for h‐*cis*PT to engage with the ER membrane, making substantial membrane contacts via both N‐terminal and other exposed aromatic residues on an adjacent helix. In this orientation, the non‐polar prenyl product is oriented correctly to potentially squeeze between the helices and insert into the adjacent ER membrane. However, this remains speculative and in need of further study, as spontaneous product insertion has not been observed in this work. Future simulations can probe this possibility more directly, building on the bound orientation developed here to develop further insight into the product release question in h‐*cis*PT.

## AUTHOR CONTRIBUTIONS

**Duncan M. Boren:** Software; writing – original draft; investigation; methodology; visualization; conceptualization; validation. **Shiri Kredi:** Investigation; visualization. **Ekaterina Positselskaya:** Investigation; visualization. **Moshe Giladi:** Conceptualization; methodology; writing – review and editing; supervision. **Yoni Haitin:** Conceptualization; methodology; writing – review and editing; supervision. **Josh V. Vermaas:** Writing – review and editing; conceptualization; software; supervision; validation.

## CONFLICT OF INTEREST STATEMENT

The authors declare no conflicts of interest.

## Supporting information


**Data S1.** Supporting Information.

**Video S1.** Supporting Information.

## Data Availability

The data that support the findings of this study are openly available in zenodo at https://zenodo.org/records/14532760.
