# Supplementary material for: Identifying and quantifying membrane interactions of the protein human cis‐prenyltransferase
Source: Protein Sci. 2025 May 24;34(6):e70167. doi: 10.1002/pro.70167 (PMC12102762; doi:10.1002/pro.70167)
Supplement: Supplementary file 1 — Data S1. Supporting Information. [file PRO-34-e70167-s002.pdf]

Supporting Information: Identifying and Quantifying Membrane Interactions of the Protein Human *cis*-Prenyltransferase

Duncan M. Boren,† Shiri Kredi,‡ Ekaterina Positselskaya,‡ Moshe Giladi,*,‡ Yoni Haitin,*,‡ and Josh V. Vermaas*,†

†*MSU-DOE Plant Research Laboratory and Department of Biochemistry and Molecular Biology, Michigan State University, East Lansing, MI USA*

‡*Department of Physiology and Pharmacology, Faculty of Medical and Health Sciences, Tel Aviv University, Tel Aviv, Israel*

E-mail: moshegil@post.tau.ac.il; yhaitin@tauex.tau.ac.il; vermaasj@msu.edu

Animation 1 By extending the prenyl substrate to a substantial, but physiologically accurate length of 20 subunits, we were able to build a system with a starting position in which the prenyl substrate was inserted into both h-*cis*PT and the ER membrane. From this pose, we simulated five replicates for 1 μs each. Across these replicas, the substrate did not dissociate from either protein or membrane. In this animation, we show the membrane (spheres colored by atom identity, hiding hydrogens) along with the nascent prenyl chain (yellow) and the heterodimer of interest to highlight the nature of this interaction.

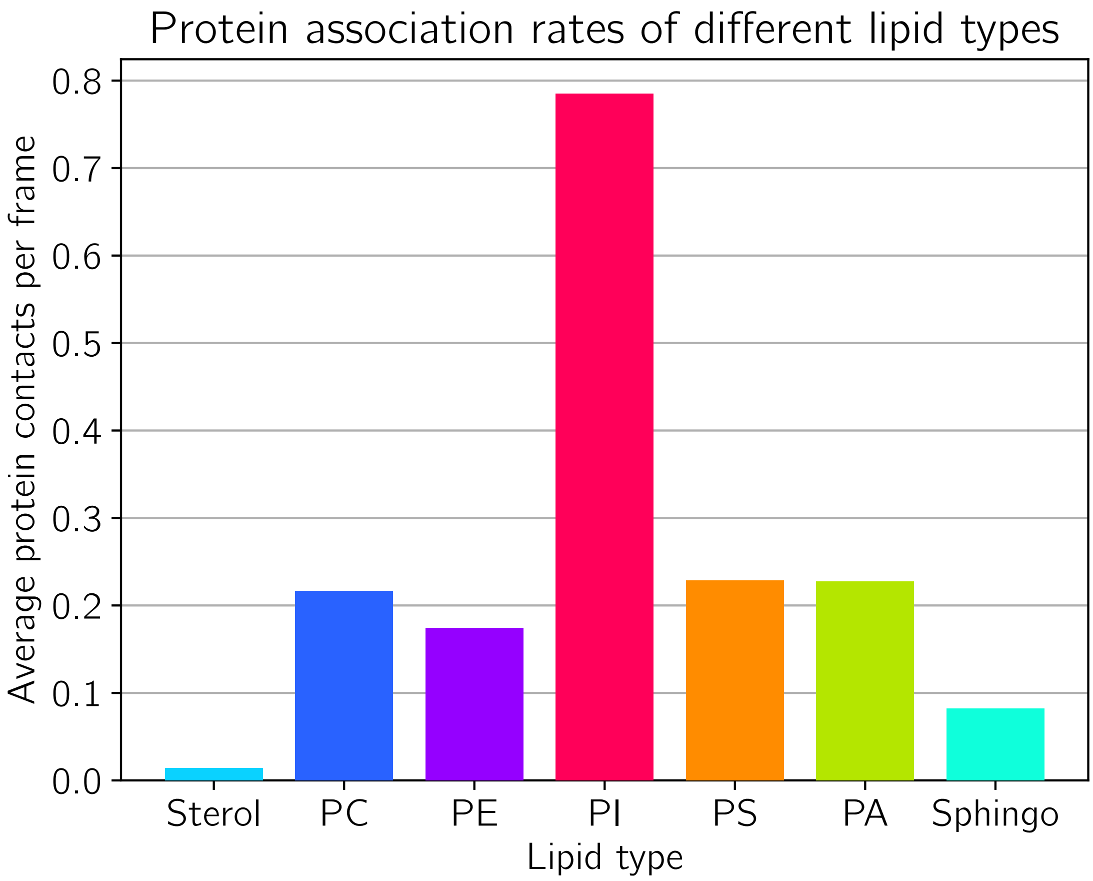

Figure S1: h-*cis*PT preferentially associates to some lipids. Contacts per frame obtained by measuring the mean contacts per frame for each lipid type, across all simulations, such that a value of 1 would indicate an average of 1 contact between lipid heavy atoms and protein heavy atoms across all 6 replicas. h-*cis*PT can form more than 1 simultaneous contact with a single lipid molecule, so frequency of association cannot be derived from this data.

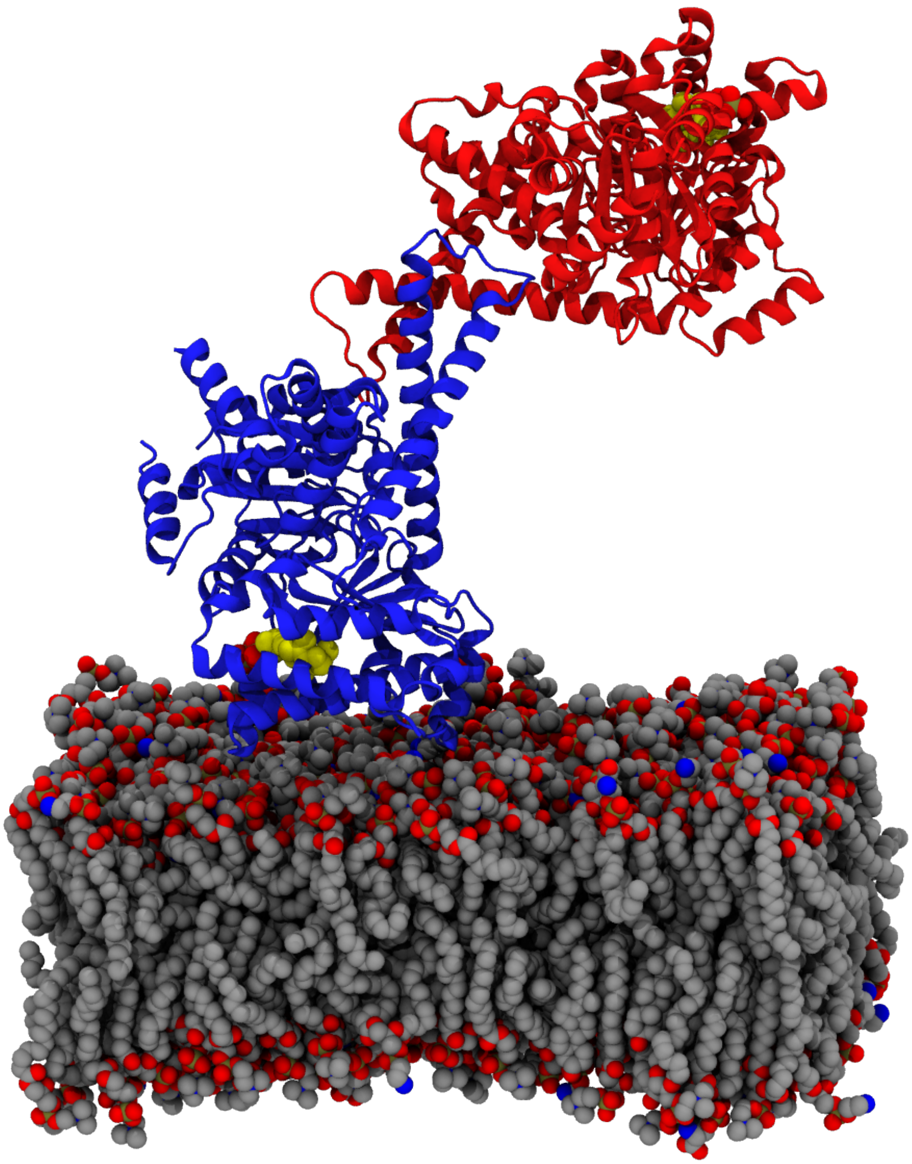

Figure S2: Heterotetrameric configuration of h-*cis*PT, taking our membrane bound pose and adding on the complementary heterodimer to form a heterotetramer based on the 7PAY PDB structure. The heterodimer originally bound to the membrane is drawn in blue, while the placed heterodimer is drawn in red, far from the membrane surface.

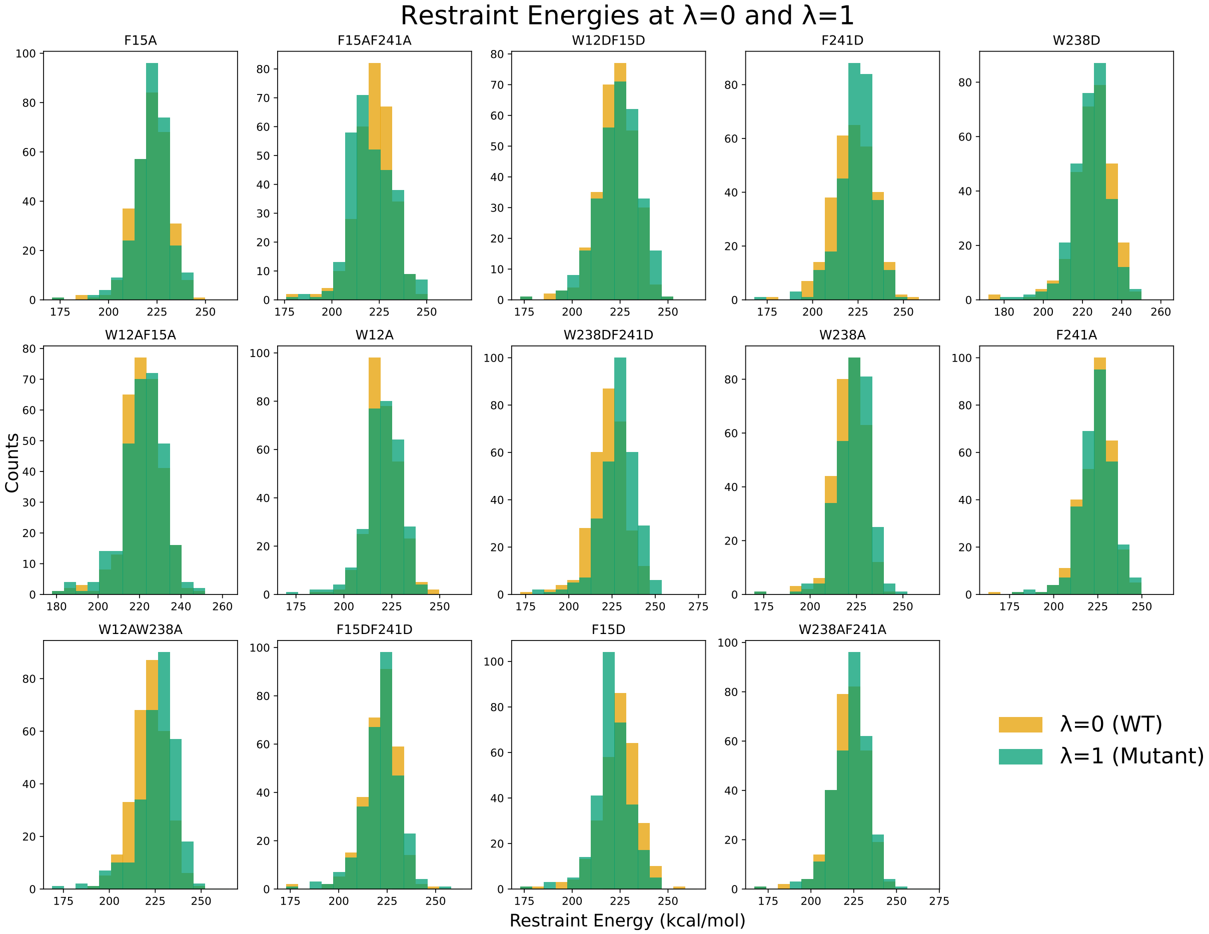

Figure S3: Energies associated with protein restraints at the beginning and end of FEP simulations, as reported by the BOUNDARY term in the NAMD log file. Each histogram shows the frequency with which a certain value (measured in kcal/mol) was output as the energy associated with maintaining the protein restraints in simulation at specific values for λ. Energy values were taken from both forward and backward FEP simulations of membrane-associated h-*cis*PT, as the difference was between the states was negligible.

Mutation	Direction	Restraint Energy Shift
F15A	forward	11.4
F15A	backward	-9.2
F15AF241A	forward	12.3
F15AF241A	backward	-13.8
W12DF15D	forward	8.9
W12DF15D	backward	-10.5
F241D	forward	8.1
F241D	backward	-10.5
W238D	forward	6.6
W238D	backward	-9.0
W12AF15A	forward	10.7
W12AF15A	backward	-9.8
W12A	forward	14.8
W12A	backward	-9.2
W238DF241D	forward	9.8
W238DF241D	backward	-0.6
W238A	forward	13.6
W238A	backward	-12.3
F241A	forward	7.1
F241A	backward	-7.0
W12AW238A	forward	15.5
W12AW238A	backward	-2.7
F15DF241D	forward	9.0
F15DF241D	backward	-12.6
F15D	forward	9.0
F15D	backward	-12.0
W238AF241A	forward	8.5
W238AF241A	backward	-11.1

Figure S4: Changes in mean restraint energy when transforming from $\lambda = 0$ and $\lambda = 1$ in forward simulations, or from $\lambda = 1$ to $\lambda = 0$ in backward simulations. This is taken from the mean of the ”BOUNDARY” term, which is where additional restraints are added to a NAMD log file.

Table S1: Primer list. Mutations were sequentially introduced using PCR. Primers were designed using the NEBaseChanger tool (nebasechanger.neb.com) (mismatched base pairs in lowercase). The F241A/D mutation was introduced first, followed by the addition of W238A/D.

Construct	Primer name	Sequence
F241A	Forward	TTGGAACCTGgcgGAAGCGATTCTGC
F241A	Reverse	AACGTATATTCCGGCCAC
F238AF241A	Forward	TGCGAACCTGgcCGAAGCGATTC
F238AF241A	Reverse	AACGTATATTCCGGCCAC
F241D	Forward	AACGTATATTCCGGCCAC
F241D	Reverse	TTGGAACCTGgatGAAGCGATTCTG
F238DF241D	Forward	TCCGGCCACAGGACCGGT
F238DF241D	Reverse	ATATACGTTTgatAACCTGGATGAAGCGATTCTG
